# Persistent SARS-CoV-2 Alpha Variant Infection in Immunosuppressed Patient, France, February 2022

**DOI:** 10.3201/eid2807.220467

**Published:** 2022-07

**Authors:** Slim Fourati, Guillaume Gautier, Myriam Chovelon, Alexandre Soulier, Melissa N’Debi, Vanessa Demontant, Céline Kennel, Christophe Rodriguez, Jean-Michel Pawlotsky

**Affiliations:** Hôpital Henri Mondor (AP-HP), Université Paris-Est, Créteil, France (S. Fourati, A. Soulier, M. N’Debi, V. Demontant, C. Rodriguez, J.-M. Pawlotsky);; William Morey General Hospital, Chalon-sur-Saône, France (G. Gautier, M. Chovelon, C. Kennel)

**Keywords:** COVID-19, SARS-CoV-2, respiratory infections, severe acute respiratory syndrome coronavirus 2, SARS, coronavirus disease, zoonoses, viruses, coronavirus, mutations, Alpha variant, France

## Abstract

We describe persistent circulation of SARS-CoV-2 Alpha variant in an immunosuppressed patient in France during February 2022. The virus had a new pattern of mutation accumulation. The ongoing circulation of previous variants of concern could lead to reemergence of variants with the potential to propagate future waves of infection.

Immunosuppressed patients can have prolonged SARS-CoV-2 infection (*1*). Studies have reported the occurrence and selection of multiple mutations in the spike glycoprotein sequence in immunosuppressed patients with persistent SARS-CoV-2 infections (*2*–*6*). To date, intrahost mutations have been described essentially in the ancestral wild-type SARS-CoV-2 virus (*3*,*5*–*8*), especially during prolonged infection with variants of concern (VOCs) (*9*). Additional SARS-CoV-2 mutations in immunocompromised persons could enable increased virus transmissibility and immune evasion, shaping the emergence of new VOCs. We describe a new mutation accumulation pattern in SARS-CoV-2 Alpha virus in an immunosuppressed patient.

An 84-year-old woman with evolutive mantle cell lymphoma who was receiving maintenance rituximab and lenalidomide treatment was admitted to the hospital on May 17, 2021. She had asthenia, fever, and hypoxia (93% oxygen saturation). At admission (day 0), she tested positive for SARS-CoV-2 RNA (Figure). She had received 2 vaccine doses 84 and 66 days before admission. She did not have respiratory symptoms, but a chest computed tomography scan showed ground-glass opacities in her lungs. The patient was hospitalized and treated with corticosteroids for 10 days. She tested SARS-CoV-2–positive again on August 26, day 101 after her initial positive test. On September 27 (day 133), she tested SARS-CoV-2–negative and was considered virologically cured. She received a vaccine booster (third) dose at day 164 and a fourth dose on day 201, but we did not detect spike receptor binding domain antibodies at days 133, 201, or 210.

**Figure:**
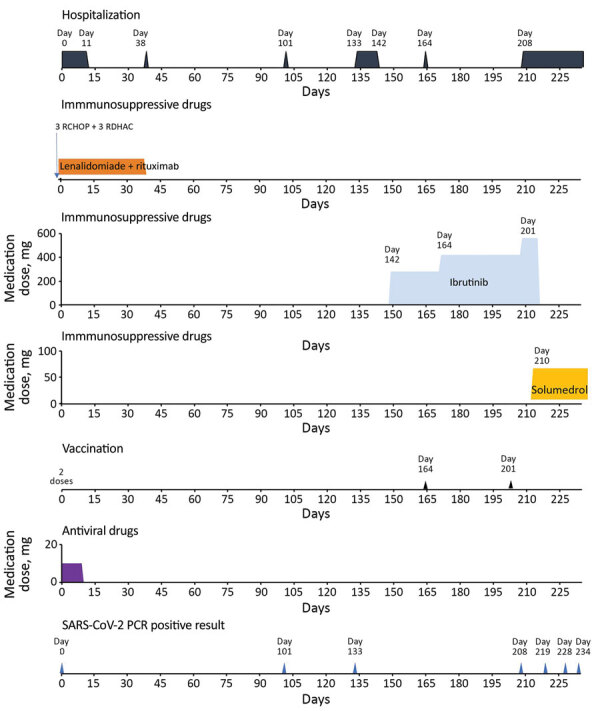
Timeline of SARS-CoV-2 diagnostic tests, hospitalizations, booster vaccination, and treatments for an immunosuppressed patient with persistent SARS-CoV-2 Alpha variant infection, France, 2022. RCHOP, combination therapy of rituximab, cyclophosphamide, doxorubicine, vincristine, and prednisone; RDHAC, combination therapy of rituximab, cytarabine, dexamethasone, and carboplatine.

Beginning in October 2021, the patient experienced intermittent fever and diarrhea because her lymphoma progressed, as shown by an abdominal computed tomography scan; she was started on a second-line treatment with ibrutinib (Figure). She was hospitalized again in early January 2022 (day 208) after clinicians discovered an evolutive lymph node mass. At admission, she tested SARS-CoV-2–positive despite the absence of COVID-19 symptoms. SARS-CoV-2 RNA levels from nasopharyngeal swab samples were high; cycle threshold values were 19 on day 208, 18 on day 228, and 22 on day 234.

At days 0, 101, 208, and 234, multiplex mutation-specific reverse transcription PCR revealed the absence of spike amino acid mutations E484Q, E484K, and L452R, and K417N was not detected on days 208 and 234, suggesting that the patient was not infected with Delta or Omicron variants, the 2 dominant variants in France at the time. These results suggested that the patient was infected with the Alpha variant, which was the dominant variant circulating when she first tested positive.

To determine whether the patient was infected with a new strain or reinfected with the same persistently replicating variant, we performed whole-genome sequencing on samples collected on days 208 (January 11, 2022) and 228 (January 31, 2022) and identified lineage B.1.1.7 (Alpha variant). Older samples were not available for sequencing. Our analysis revealed the presence of amino acid substitutions and mutations in addition to characteristics of the Alpha variant, including mutations in open reading frame (ORF) 1a (nonstructural protein 3, n = 4), spike protein (n = 6), matrix protein (n = 1), envelope protein (n = 2), ORF3a (n = 1), and ORF7 (n = 2) (Appendix Figure).

The SARS-CoV-2 mutational pattern in this immunosuppressed patient adds several new mutations to the Alpha variant characteristic. Although earlier samples were not available for sequencing, mutations in later samples align with an ongoing selection process. The mutations we observed share similarities with those observed in other VOCs and variants of interest, pointing to evolutionary convergence, such as spike del241–247, which also is found in part in the Beta variant. Several mutations that likely play a role in immune evasion were selected in the spike nucleocapsid terminal domain (e.g., K77E, S248F, and del14–18) and receptor-binding domain (L452M). These mutations have rarely been reported in isolates submitted to GISAID (https://www.gisaid.org), suggesting that, when considered individually, they could be maladaptive.

In January 2022, the Alpha variant was no longer circulating in France, according to strains submitted to GISAID. Our case highlights the potential for persistence of supposedly extinct SARS-CoV-2 variants that might cause prolonged infection in immunocompromised patients and acquire adaptive mutations that confer increased transmissibility, antigenic divergence, and reduced pathogenicity, with obvious public health implications (*1*,*3*). Similar cases likely exist in other parts of the world because SARS-CoV-2 genome sequencing and reporting to GISAID are far from exhaustive.

Omicron-infected patients not immunized against older variants appear to mount a weak or no neutralizing response against variants that preceded Omicron, including VOCs (R.K. Suryawanshi et al., unpub. data, https://doi.org/10.1101/2022.01.13.22269243). Because the next dominant variant could emerge from a variant other than Omicron, ongoing circulation of older VOCs could feed reemergence of variants that eliminate Omicron, particularly in unvaccinated populations, emphasizing the crucial role of vaccination to prevent new SARS-CoV-2 waves.

In conclusion, this report highlights the need to reinforce precautions to avert nosocomial and community transmission involving immunocompromised patients, who might shed older SARS-CoV-2 variants longer. Prospective genomic surveillance for SARS-CoV-2 variants is needed in persons with prolonged infection, particularly in countries with many immunocompromised persons, such as countries with a high HIV prevalence and low vaccination rates.

Appendix Additional methods used to investigate persistent SARS-CoV-2 Alpha variant in an immunocompromised patient, France, 2022.
