# Supplementary material for: Persistent SARS-CoV-2 Alpha Variant Infection in Immunosuppressed Patient, France, February 2022
Source: Emerg Infect Dis. 2022 Jul;28(7):1512–5. doi: 10.3201/eid2807.220467 (PMC9239896; doi:10.3201/eid2807.220467)
Supplement: Appendix — Additional methods used to investigate persistent SARS-CoV-2 Alpha variant in an immunocompromised patient, France, 2022. [file 22-0467-Techapp-s1.pdf]

Article DOI: https://doi.org/10.3201/eid2807.220467

Persistent SARS-CoV-2 Alpha Variant Infection in Immunosuppressed Patient, France, February 2022

Appendix

Methods

We used GSD NovaType IV SARS-CoV-2 (Gold Standard Diagnostics, https://www.goldstandarddiagnostics.com), a multiplex mutation-specific reverse transcription PCR (RT-PCR) kit based on melting curve analyses, to search for the presence of Spike amino acid mutations E484Q, E484K, L452R, and beginning in January 2022, for K417N.

Full-length SARS-CoV-2 genome sequence analysis was performed from nasopharyngeal swab samples by using the COVIDSeq Test (Illumina, https://www.illumina.com). The sequences were demultiplexed and assembled as full-length genomes with the DRAGEN COVIDSeq Test Pipeline on a DRAGEN server (Illumina). Lineages were interpreted with Pangolin (https://github.com/cov-lineages/pangolin) and clades with Nextclade (Nextstrain, https://clades.nextstrain.org). All sequences were submitted to GISAID (https://www.gisaid.org) database and to GenBank (https://www.ncbi.nlm.nih.gov/genbank) under accession no. SUB11349926.

Our study protocol followed the ethical guidelines of the Declaration of Helsinki and was approved by our institutional review board. The patient provided informed consent.

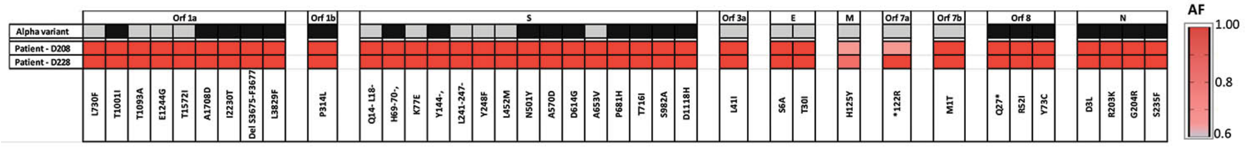

Appendix Figure. SARS-CoV-2 genome mutational patterns relative to an Alpha variant consensus in an immunosuppressed patient with persistent SARS-CoV-2 Alpha variant infection, France, 2022. Sequence data are from nasopharyngeal samples collected at days 208 and 228. Black boxes indicate Alpha variant–defining mutations according to GISAID (https://www.covariants.org/variants/20I.Alpha.V1). Grey boxes indicate mutations other than those in the Alpha variant sequence. Red boxes indicate mutations in the patient’s sequences at days 208 and 228. AF, allele frequency; E, extinction gene; M, matrix gene; N, nucleocapsid gene; ORF, open reading frame; S, spike gene.
